# Observations upon the Treatment of Some Cases of Neurasthenia

**Published:** 1898-08

**Authors:** Jerome K. Bauduy

**Affiliations:** St. Louis, Mo., Professor Nervous and Mental Diseases, and of Medical Jurisprudence, Missouri Medical College


### Abstracts and Selections.

### Observations Upon the Treatment of Some Cases of Neurasthenia.

BY JEROME K. BAUDUY, M. D., ST. LOUIS, MO.,
Professor Nervous and Mental Diseases, and of Medical Jurisprudence, Missouri Medical College.

Clinical Report by Keating Bauduy, M. D., St. Louis, Mo. Microscopic Report by C. Fisch, M. D., St. Louis, Mo.

Read before the St. Louis Medical Society, Saturday Evening, February 5, 1898.

That chalybeates, more especially the *organic* salts of iron, constitute an essential indication in the successful treatment of some cases of neurasthenia, especially in the female, where functional menstrual derangements exist, is to my mind *an indisputable fact.* They produce conditions, oftentimes not attainable by the inorganic preparations for many reasons, which experience and reflection clearly demonstrate.

In a recent clinical study of this affection, my conclusion, as above stated, is fully justified and corroborated by the microscopical blood examinations conducted by my esteemed and skillful friend, Dr. C. Fisch. That cerebro-spinal anæmia is a frequent important concomitant, if not an essential etiological factor of neurasthenia, I hardly think admits of cavil.

The clinical histories of appended cases were compiled by my son, Dr. Keating Bauduy, chief of the Neurological Clinic at St. John's Hospital, under whose direct supervision the investigations were conducted. That the ratio, or number of red blood corpuscles, and the percentage of hæmoglobin were deficient in the normal standard of these cases, prior to the treatment, *is incontestable*, as shown by the microscope. That several of the cases to be enumerated showed marked improvement, even after one or two weeks treatment, is moreover revealed in the same manner, and which for rapidity of effect is quite an exceptional, if not a startling therapeutic result, when compared with some of the prior and more established methods of treatment. That many of these cases presented unmistakable evidence of satisfactory improvement, from both a subjective and objective standpoint, was quite as notable as the permanent character of their general amelioration. That the ordinary tonic had in some instances been administered with nugatory results, while pursued along the old lines of authoritative medication, seems quite manifest.

My only explanation of the *surprising results* in the cases herein cited, where the usual officinal class of remedies had formerly been ineffectually essayed, was the superinduction as is so frequently the case of disturbed digestion and assimilation; results but too familiar and disappointing to professional experience. Aside from the disturbances just mentioned, the development of headache, constipation, etc., frequently obviates their further administration.

When, a few years ago, my attention was called to Gude's preparation of "*Liquor Mangano-Ferri Peptonatus*, *Gude*," (Pepto-Mangan) so extensively used and highly extolled in Germany, with my usual antipathy for new remedies, I reluctantly gave it a trial, anticipating that I would necessarily have to combat the usual disappointing effects of the other preparations of iron. The results, however, were *indeed a surprise to myself*, for the concomitant deranging sequelæ were so slight, that but in very few instances in *my extensive* ultilization and experience with this special pharmaceutical preparation was I obliged to discontinue it. My experience having led me to believe that iron and manganese in combination are both indicated in the vast majority of cases of neurasthenia, this particular remedy, *I am now convinced*, *will prove a great boon both to the patient and the physician*. While it is maintained by some that in the hæmoglobin of the red blood corpuscle manganese is present, as well as iron, I have for many years produced results with a combination of both, not directly obtainable with one alone. We know, however, that manganese gives off oxygen to a greater degree than iron, and it has been argued that for this reason its internal exhibition might correspondingly increase assimilation.

Dr. Fisch's appended microscopical report shows that the increase in the percentage of hæmoglobin, in many of this series of cases, is far in excess of the proportionate increase of the red blood corpuscles. *This fact I deem of greater importance as to the effectiveness of the medicine*, because the count of the blood corpuscles is to a certain extent relative, and the size varies greatly in different cases, and for other reasons the same amount of blood plasma contains different numbers of red cells; hence I would particularly lay stress upon the proportionate increase of the hæmoglobin as the more important factor. The *notable* and *astonishing improvement* of these cases, when placed upon this preparation, led me to their closer scrutiny, as well as microscopic observation. Before concluding, I *wish particularly* to call attention to the fact of the absence of digestive disturbances and necessary consequent interference in the assimilation. All other unpleasant complicating results were notable by their absence. Of course we do not consider the remedy applicable to cases of lithemic neurasthenia, nor in any way a *specific* in any variety of neurasthenia. In many cases the addition of arsenic and strychnia greatly increases the efficacy of the preparation. I must also take cognizance of the salient fact of the rapidity with which a large number of female neurasthenics, under our treatment, who have suffered with marked functional menstrual derangements, have attained a normal condition under the administration *of this most elegant combination of iron and manganese.*

As it would be tedious and monotonous to present an exhaustive citation of multiplicity of clinical cases, I have confined myself to a recital of a few typical ones.

CLINICAL REPORT—BY KEATING BAUDUY, M. D.

CASE 1.—Mrs. S, aged 32 years, mother of three children, came to me in a pitiable mental condition, and had in her arms a nursing hydrocephalic child, five months old. Her mental depression approached a type of veritable melancholia. My first idea was to advise that the child be weaned, and then place her upon the classical opium treatment for melancholia. This was her third child, and like all mothers, she clung to the life of her unfortunate with characteristic tenderness. Therefore she bluntly insisted upon my candid opinion, as to whether the weaning of the baby might prove fatal. Knowing, as I did, that the life of the child was simply a question of a period of short duration in either case, I so informed her; nevertherless,

I insisted that the best hope for her recovery was to wean it. This she refused to do, and after Dr. Fisch had made a blood examination and pronounced her highly anæmic, I reluctantly undertook the case. Aside from her mental depression, physical lassitude, and marked pallor, the "casque neurasthenique" symptom was a dominant feature in her case. Any effort to perform her usual household duties produced sensations of cerebral fullness, and persistent pain in the vertex. She even confessed that the idea of suicide had of late frequently haunted her. Under the administration of "Pepto-Mangan," with no other treatment, after the short period of fifty-two days, she was discharged fully restored to her normal condition. Microscopic reports showed a relative gain in red blood corpuscles of 34 per cent.; hemoglobin, 44.5 per cent.

Case 1.—I. Examination (Beginning of treatment), date, November 17, 1897: Red corpuscles, 3,120,000; hemoglobin, per cent., 52. II. Examination, date, December 2, 1897: Red corpuscles, 3,400,000; hemoglobin, per cent., 54. III. Examination, date, December 26, 1897: Red corpuscles, 4,130,000; hemoglobin, per cent., 67. IV. Examination, date, January 8, 1898: Red corpuscles, 4,245,000; hemoglobin, per cent., 75. Duration of treatment 52 days. Gain (absolute): Red corpuscles (in 1000's), 1125; hemoglobin, per cent., 23. Gain (relative): Red corpuscles, per cent., 34; hemoglobin, per cent., 44.5.

Case 2.—Mrs. Sim, aged 23 years, mother of two children, youngest six months and nursing. About the fourth month of her last pregnancy she was troubled with dyspnœa. Gave history of instrumental delivery, followed by puerperal eclampsia. Great loss of blood during birth of child. Two months later, abscesses developed in each breast, and patient was confined to bed during a period of ten weeks. Case presented typical manifestations of neurasthenia; also characteristic apprehensions, with preternatural emotional mobility. Constant cephalgia in vertical region, persistent parasthesiæ in extremities, mouth and tongue, were also present. She was intensely pale with every appearance of profound anæmia. Aside from a mild laxative which was given to obviate constipation—an obstinate feature in her case—nothing was administered, save "Pepto-Mangan." After a period of treatment of forty-nine days I discharged her, as she evinced none of the symptoms which formerly existed. A notable feature was the corresponding improvement of the child, notwithstanding the fact that I had previously insisted upon its being weaned, which she had, nevertheless contrary to my instructions, continued to nurse. Microscopic report showed a relative gain: red blood corpuscles 19 per cent.; hemoglobin 27 per cent.

Case 2.—I. Examination (beginning of treatment), date, November 20, 1897: Red corpuscles, 3,470,000; hemoglobin, per cent., 60. II. Examination, date, December 22, 1897: Red corpuscles, 3,980,000; hemoglobin, per cent., 69. III. Examination, date, January 8, 1898: Red corpuscles, 4,120,000; hemoglobin, per cent., 76. Duration of treatment, 40 days. Gain (absolute): Red corpuscles (in 1000's), 650; hemoglobin, per cent., 16. Gain (relative): Red corpuscles, per cent, 19; homoglobin, per cent., 27.

Case 3.—D. G., aged 25 years, unmarried. Suffered from nervous headache for past year. Vaso-motor disturbances evidenced by alternate flushings and pallors, heat and cold. Atonic dyspepsia. Irregularity of bowels. Disturbed sleep. Depressed physical condition, correspondingly weak pulse. After taking "Pepto-Mangan" fifty-seven days, reported feeling greatly improved. Digestion was better, pulse stronger and headaches greatly diminished in intensity. Vaso-motor disturbances disappeared. Microscopic examination showed a relative gain: red blood corpuscles 11 per cent.; hemoglobin 15 per cent.

Case 3.—I. Examination (beginning of treatment), date, November 26, 1897: Red corpuscles, 3,720,000; hemoglobin, per cent., 61. II. Examination, date, January 22, 1898: Red corpuscles, 4,135,000; hemoglobin, per cent., 70. Duration of treatment, 57 days. Gain (absolute): Red corpuscles (in 1000's), 415; hemaglobin, per cent., 9. Gain (relative): Red corpuscles, per cent., 11; hemoglobin, per cent., 15.

Case 4.—Miss S., aged 28 years, presenting many of the well-defined symptoms of neurasthenia, was in a condition of profound mental and physical weakness. The history showed that since our great cyclone of May 27, 1896, she had never been her normal self, and was unable to perform any sustained mental or physical strain. Dating from that episode she had always worried, and was constantly the victim of peculiar forebodings. Insomnia and general malaise were cardinal symptoms. My diagnosis was what has been termed "cyclone neurosis," of which I have seen numerous cases. Menorrhagi a e x isted to an alarming extent, for which I accordingly recommended rest and the recumbent position during her periods. Because of the pronounced insomnia, I prescribed a nightly dose of hyoscyamine and sulfonal during the first week of treatment as a hypnotic, which constituted the only medication other than "Pepto-Mangan." After having taken the latter for forty-one days, I discharged her from treatment, as she had passed her last menstrual period after a normal flow of three days, her pallor giving way to rosy cheeks and her physical and mental condition being entirely satisfactory. Microscopic report showed a relative gain: red blood corpuscles 38 per cent.; hemoglobin 47 per cent.

Case 4.—I. Examination (beginning of treatment), date, November 26, 1897: Red corpuscles, 2,807,000; hemaglobin, per cent., 46. II. Examination, date, December 17, 1897: Red corpuscles, 3,200,000; hemoglobin, per cent., 50. III. Examination, date, January 4, 1898: Red corpuscles, 3,250,000; hemoglobin, per cent., 56. IV. Examination, date, January 8, 1898: Red corpuscles, 3,875,000; hemoglobin, per cent., 68. Duration of treatment, 41 days. Gain (absolute): Red corpuscles (in 1000's), 1968; hemoglobin, per cent., 22. Gain (relative): Red corpuscles, per cent., 38; hemoglobin, per cent., 47.

Case 5.—Mr. C., aged 21 years, unmarried. Highly anæmic, very pale. Anorexia and insomnia persistent. Physical condition greatly depressed. Cardinal feature was a sexual hypochondriacal tendency. Gave history of excesses both alcoholic and sexual. Aside from advice as to the necessity of leading a moral life, and abstaining from all stimulants, gave no medicine but "Pepto-Mangan," with the addition of arsenic and strychnia. After fifty-seven days of treatment, patient was much benefited. Microscopic report showed a relative gain: Red blood corpuscles 9 per cent.; hemoglobin 27 per cent.

Case 5.—I. Examination (beginning of treatment), date, November 26, 1897: Red corpuscles, 3,670,000; hemoglobin, per cent., 44. II. Examination, date, December 14, 1897: Red corpuscles, 3,700,000; hemaglobin, per cent., 42. III. Examination, date, January 8, 1898: Red corpuscles, 3,990,000; hemoglobin, per cent., 54. IV. Examination, date, January 22, 1898: Red corpuscles, 4,010,000; hemoglobin, per cent., 56. Duration of treatment, 57 days. Gain (absolute): Red corpuscles (in 1000's), 340; hemoglobin, per cent., 12. Gain (relative): Red corpuscles, per cent., 9; hemoglobin, per cent., 27.

CASE 6.—Mrs. D., aged 36 years, married; five children. Since birth of last child, eighteen months ago, has been in state of profound nervous prostration. Previously resisted ordinary tonic and constructive treatment. Menorrhagia was the dominant feature of the case. After taking "Pepto-Mangan" for fifty-one days patient evinced more improvement than during any stated time throughout the past eighteen months. Last menstruation approached the normal flow. Microscopical report showed a relative gain: red blood corpuscles 13 per cent., hemoglobin 8 per cent.

Case 6.—I. Examination (beginning of treatment), date, November 26, 1897: Red corpuscles, 3,450,000; hemoglobin, per cent., 60. II. Examination, date, December 22, 1897: Red corpuscles, 3,820,000; hemoglobin, per cent., 62. III. Examination, date, January 8, 1898: Red corpuscles, 3,916,000; hemoglobin, per cent., 62. IV. Examination, date, January 16, 1898: Red corpuscles, 3,890,000; hemoglobin, per cent., 65. Duration of treatment, 51 days. Gain (absolute): Red corpuscles (in 1000's), 440; hemoglobin per cent., 5. Gain (relative): Red corpuscles, per cent., 13; hemoglobin, per cent., 8.

CASE 7.—Mrs. J., aged 48 years, widow; mother of a large family. Cardinal feature of case was recurrent cephalalgia at intervals of several days. This case reported an improvement as to the intensity and duration of headaches, after the period of fourteen days of treatment. Only two blood examinations were made. A further opportunity to observe this patient did not present itself, in consequence of her failure to continue the treatment. Microscopic examination showed a relative gain: red blood corpuscles 14 per cent.; hemoglobin 13 per cent.

Case 7.—I. Examination (beginning of treatment), date, November 30, 1897: Red corpuscles, 3,210,000; homoglobin, per cent., 60. II. Examination, date, December 14, 1897: Red corpuscles, 3,670,000; hemoglobin, per cent., 68. Duration of treatment, 14 days. Gain (absolute): Red corpuscles (in 1000's), 460; hemoglobin, per cent., 8. Gain (relative): Red corpuscles, per cent., 14; hemoglobin, per cent., 13.

CASE 8.—H. F., aged 18 years, school teacher, unmarried. Symptomatology of neurasthenia. Malaria was a complicating feature. Amenorrhœa for six months was the principal symptom for which she consulted me. Aside from a course of quinine to eradicate the malarial feature, I gave exclusively "Pepto-Mangan." After forty-seven days treatment she was apparently much improved, her menses having appeared in the interim. Microscopic examination showed a relative gain: red blood corpuscles 9 per cent.; hemoglobin 22 per cent.

Case 8.—I. Examination (beginning of treatment), date, November 30, 1897: Red corpuscles, 2,970,000; hemoglobin, per cent., 42. II. Examination, date, January 8, 1898: Red corpuscles, 3,100,000; hemoglobin, per cent., 49. III. Examination, date January 16, 1898: Red corpuscles, 3,250,000; hemoglobin, per cent., 51. Duration of treatment, 47 days. Gain (absolute): Red corpuscles (in 1000's), 280; Hemoglobin, per cent., 9. Gain (relative): Red corpuscles, per cent., 9; hemoglobin, per cent., 22.

Case 9.—Mrs. L., aged 42 years, married, three children. Comes from neuropathic family, one uncle an epileptic. Has always been quite delicate and anæmic. Since sudden death of husband has manifested great irritability of temper. Loses control of herself upon the slightest provocation. Cries easily, but not melancholic. Peculiarly apprehensive of sudden death; imagines upon retiring, that she will never awake; paroxysmal attacks of anxiety, and fatigued upon the slightest exertion. Anorexia. Habitual constipation. Sleeps restlessly. Patient although still very pale, after taking "Pepto-Mangan" for twenty-seven days, began to manifest a general improvement. Microscopic report showed a relative gain: red blood corpuscles 11 per cent., hemoglobin 12 per cent.

Case 9.—I. Examination (beginning of treatment), date, January 2, 1898: Red corpuscles, 3,720,000; hemoglobin, per cent., 54. II. Examination, date, January 22, 1898: Red corpuscles, 4,105,000; hemoglobin, per cent., 60. III. Examination, date, January 29, 1898: Red corpuscles, 4,130,000; hemoglobin, per cent., 64. Duration of treatment, 27 days. Gain (absolute): Red corpuscles (in 1000's), 410; hemoglobin, per cent., 10. Gain (relative): Red corpuscles, per cent., 11; hemoglobin, per cent., 12.

Case. 10.—Mrs. P., aged 36 years, married, no children. Family history predisposed to tuberculosis. Physically in good health. Since cyclone, May 27, 1896, when her house was totally destroyed, and she narrowly escaped death, she developed nervous headaches; later on she manifested a listless and apathetic condition. Sleeps excellently, but does not feel refreshed upon awakening. Complains of drowsiness. Marked irritability of temper. Appetite fair, but nervous dyspepsia. Boards with sister, as she cannot muster courage to manage a household of her own. After taking "Pepto-Mangan" for twenty-five days she began to feel much brighter and better, but still occasional lapses into her former indifferent mood. Color better, and nervous dyspepsia greatly relieved. Microscopic report showed a relative gain: red blood corpuscles 12 per cent; hemoglobin 12 per cent.

Case 10.—I. Examination (beginning of treatment), date, January 4, 1898: Red corpuscles, 3,124,000; hemoglobin, per cent., 56. II. Examination, date, January 14, 1898: Red corpuscles, 3,200,000; Hemoglobin, per cent., 57. III. Examination, date, January 22, 1898: Red corpuscles, 3,250,000; hemoglobin, per cent., 62. IV. Red corpuscles, 3,460,000; hemoglobin, per cent., 68. Duration of treatment, 25 days. Gain (absolute): Red corpuscles (in 1000's), 336; hemoglobin, per cent., 12. Gain (relative): Red corpuscles, per cent., 12; hemoglobin, per cent., 12.

Case 11.—Mr. M., aged 29 years. Family history tuberculous. His avocation was that of a "book-maker" during the past few years. The strain of gambling and the consequent excitement and worry have made him a nervous wreck. Jerky and fidgety at all times. Inability to concentrate his mind at any time. Suffers from nightmares and phantasmagoria during sleep, which is consequently much disturbed. Is troubled with constipation and greatly digestion. Anorexia marked. Much reduced in weight. Although always fatigued and depressed, he constantly walks to relieve his pent-up nervous irritability. Dreads to be alone for fear something may happen to him. After the administration of "Pepto-Mangan" for twenty-four days, patient reports a general improvement, especially as to his appetite and the relief of his digestion. Microscopic report showed a relative gain: red blood corpuscles 11 per cent.; hemoglobin 12 per cent.

Case 11.—I. Examination (beginning of treatment), date, January 5, 1898: Red corpuscles, 3,856,000; hemoglobin, per cent., 63. II. Examination, date, January 14, 1898: Red corpuscles, 4,001,000; hemoglobin, per cent., 65. III. Examination, date, January 22, 1898: Red corpuscles, 4,051,000; hemoglobin, per cent., 71. IV. Examination, date, January 29, 1898: Red corpuscles, 4,120,000; hemoglobin, per cent., 75. Duration of treatment, 24 days. Gain (absolute): Red corpuscles (in 1000's),

264; hemoglobin, per cent., 12. Gain (relative): Red corpuscles, per cent., 11; hemoglobin, per cent., 12.

CASE 12.—A. McG., aged 20 years, servant, unmarried. History showed the ordinary "symptom-group" of neurasthenia. After the short period of seven days, having taken but one bottle of "Pepto-Mangan," her condition was greatly alleviated. Microscopic report showed a relative gain: red blood corpuscles, 5 per cent.; hemoglobin, 8 per cent.

Case 12.—I. Examination (beginning of treatment), date, January 16, 1898: Red corpuscles, 2,985,000; hemoglobin, per cent., 49. II. Examination, date, January 23, 1898: Red Corpuscles, 3,120,000; hemoglobin, per cent, 53. Duration of treasment, 7 days. Gain (absolute): Red corpuscles (in 1000's), 135; hemoglobin, per cent., 4. Gain (relative): Red corpuscles, per cent., 5; hemoglobin, per cent., 8.—From the *Medical Review*, St. Louis, Mo., February 26, 1898.

